# An updated gene atlas for maize reveals organ‐specific and stress‐induced genes

**DOI:** 10.1111/tpj.14184

**Published:** 2019-01-22

**Authors:** Genevieve M. Hoopes, John P. Hamilton, Joshua C. Wood, Eddi Esteban, Asher Pasha, Brieanne Vaillancourt, Nicholas J. Provart, C. Robin Buell

**Affiliations:** ^1^ Department of Plant Biology Michigan State University East Lansing MI 48824 USA; ^2^ Department of Energy Great Lakes Bioenergy Research Center Michigan State University East Lansing MI 48824 USA; ^3^ Department of Cell and Systems Biology/Centre for the Analysis of Genome Evolution and Function University of Toronto Toronto Ontario M5S 3B2 Canada; ^4^ Plant Resilience Institute Michigan State University East Lansing MI 48824 USA; ^5^ Michigan State University AgBioResearch East Lansing MI 48824 USA

**Keywords:** *Zea mays*, B73, AGPv4, differential expression, co‐expression, presence−absence variants, gene atlas

## Abstract

Maize (*Zea mays* L.), a model species for genetic studies, is one of the two most important crop species worldwide. The genome sequence of the reference genotype, B73, representative of the stiff stalk heterotic group was recently updated (AGPv4) using long‐read sequencing and optical mapping technology. To facilitate the use of AGPv4 and to enable functional genomic studies and association of genotype with phenotype, we determined expression abundances for replicated mRNA‐sequencing datasets from 79 tissues and five abiotic/biotic stress treatments revealing 36 207 expressed genes. Characterization of the B73 transcriptome across six organs revealed 4154 organ‐specific and 7704 differentially expressed (DE) genes following stress treatment. Gene co‐expression network analyses revealed 12 modules associated with distinct biological processes containing 13 590 genes providing a resource for further association of gene function based on co‐expression patterns. Presence−absence variants (PAVs) previously identified using whole genome resequencing data from 61 additional inbred lines were enriched in organ‐specific and stress‐induced DE genes suggesting that PAVs may function in phenological variation and adaptation to environment. Relative to core genes conserved across the 62 profiled inbreds, PAVs have lower expression abundances which are correlated with their frequency of dispersion across inbreds and on average have significantly fewer co‐expression network connections suggesting that a subset of PAVs may be on an evolutionary path to pseudogenization. To facilitate use by the community, we developed the Maize Genomics Resource website (maize.plantbiology.msu.edu) for viewing and data‐mining these resources and deployed two new views on the maize electronic Fluorescent Pictograph Browser (bar.utoronto.ca/efp_maize).

## Significance Statement

We developed a comprehensive transcriptome resource for the maize reference genotype B73 based on the AGPv4 long‐read assembly using replicated and publicly available RNA‐sequencing datasets. Gene expression analyses were performed on a robust B73 developmental and stress gene expression atlas, utilizing core and dispensable genes of the maize pan‐genome as a case study to demonstrate the utility of the data.

## Introduction

Based on tonnage, maize (*Zea mays* L.) is the most produced staple crop worldwide (http://www.fao.org/faostat). While significant advances have been made in maize improvement efforts since domestication, targeted advancements in yield and stress tolerance will be needed to address increasing population sizes and changing climates (Gong *et al*., 2015). Improving our understanding of gene function including how altered environments affect the transcriptome and the resulting phenotype will facilitate development of improved maize cultivars.

Gene expression profiling of development and abiotic/biotic stress treatments has been performed previously in maize resulting in identification of organ‐specific, differentially expressed (DE) gene sets, and co‐expression networks (Opitz *et al*., 2014; Makarevitch *et al*., 2015; Stelpflug *et al*., 2016; Huang *et al*., 2017; Swart *et al*., 2017). With respect to the B73 reference genotype, representative of the stiff stalk heterotic group, Sekhon *et al*. (2011) profiled gene expression in 60 B73 tissues using microarrays that were later quantified via RNA‐sequencing (RNA‐seq) (Sekhon *et al*., 2013), permitting a more robust assessment of gene expression among paralogs. Additional developmental sampling of the B73 genotype includes a post‐pollination leaf and internode time course (Sekhon *et al*., 2012) and a root developmental series (Stelpflug *et al*., 2016) yielding 79 replicated tissue samples from six organs that span the majority of the developmental stages and tissues that have been extensively analyzed including construction of co‐expression networks (Huang *et al*., 2017). Several high quality biotic and abiotic stress experiments have also been conducted in B73 revealing transcriptional changes under *Cercospora zeina* infection (Swart *et al*., 2017), drought stress (Opitz *et al*., 2014), and temperature stress (Makarevitch *et al*., 2015). While these analyses are available for the community, all of these analyses were performed using the previous B73 genome assembly and annotation. Recently, version 4 of the B73 genome, which was assembled from long‐reads and validated with optical mapping, and an updated set of gene annotations (AGPv4) were released (Jiao *et al*., 2017). While data are available to convert loci between deprecated versions of the B73 genome, these do not take into account changes to the underlying sequence and/or annotation in AGPv4. Indeed, 8549 v3 genes could not be mapped to the v4 annotation and an additional 68 245 filtered protein‐coding transcripts were added to the AGPv4 genome assembly (Jiao *et al*., 2017).

The pan‐genome concept, first introduced by Tettelin *et al*. (2005) in the bacterium *Streptococcus agalactiae* (Tettelin *et al*., 2005), has now been characterized in a range of plant species (Springer *et al*., 2009; Tan *et al*., 2012; Golicz *et al*., 2016; Hardigan *et al*., 2016). The pan‐genome is composed of core genes present in all accessions of a species whereas dispensable genes, structural variants in the form of copy number variants (CNVs) and PAVs are present in a subset of accessions of the species. In plants, CNVs and PAVs have been shown to function in environmental adaptation responses including flowering time (Díaz *et al*., 2012), secondary metabolism (Winzer *et al*., 2012), and stress tolerance (Hattori *et al*., 2009; Gaines *et al*., 2010; Cook *et al*., 2012). In maize, several pan‐genome studies have been conducted using array comparative genomic hybridization (Springer *et al*., 2009; Swanson‐Wagner *et al*., 2010), RNA‐sequencing (Hirsch *et al*., 2014a), whole genome resequencing (Lai *et al*., 2010; Jiang *et al*., 2015; Brohammer *et al*., 2018), genotyping‐by‐sequencing (Lu *et al*., 2015), and whole genome comparison (Hirsch *et al*., 2016; Sun *et al*., 2018). Recently, using resequencing data from 62 inbred lines with a CDS (coding sequence) coverage threshold of 20%, Brohammer *et al*. (2018) identified 30.7% of the anchored B73 v4 loci as absent in at least one inbred line; and 581 loci were absent in 41 to 61 of the inbreds (Brohammer *et al*., 2018). The pervasiveness of PAVs in the maize genome led Swanson‐Wagner *et al*. (2010) to theorize that PAVs present in gene families have functional redundancy, as other family members can minimize gene loss effects (Swanson‐Wagner *et al*., 2010). While some PAVs have been demonstrated to have a function (Hattori *et al*., 2009; Winzer *et al*., 2012), most PAVs lack a known function; gene expression studies could better associate PAVs with putative function.

Here, the 79‐tissue B73 developmental gene atlas data (Stelpflug *et al*., 2016) and five publicly available stress transcriptomic experiments (Opitz *et al*., 2014; Makarevitch *et al*., 2015; Swart *et al*., 2017) were used with the updated AGPv4 assembly and annotation of the reference accession B73 to generate a comprehensive gene atlas encompassing both development and stress responses. Differential gene expression analyses were performed to identify organ‐specific and stress‐related DE genes and weighted gene co‐expression network analysis (WGCNA) was conducted to classify genes in co‐expression modules and determine gene correlation patterns. Using these gene expression analyses, core genes and PAVs identified by Brohammer *et al*. (2018) were studied to determine the differential and co‐expression characteristics across a diverse set of maize inbreds and provide a foundational dataset to infer function. To facilitate use by the community, the data along with search and analysis tools are available via the Maize Genomics Resource (MGR; maize.plantbiology.msu.edu) and the Bio‐Analytic Resource for Plant Biology (BAR) Maize electronic Fluorescent Pictograph (eFP) Browser (bar.utoronto.ca/efp_maize).

## Results and Discussion

### Quality assessment of transcriptome data

Publicly available datasets for B73 RNA‐seq experiments that had at least three biological replicates per condition with each replicate containing at least 10 million reads were included in this study. Transcriptomic data for 222 samples for 79 tissues from six organs representing the key developmental stages in B73 (Sekhon *et al*., 2011, 2012, 2013; Stelpflug *et al*., 2016) were combined with 52 samples representing replicated data from five publicly available B73 abiotic and biotic stress experiments (Data S1); in total 274 samples were analyzed. Biotic stress experiments included leaves challenged with two fungal pathogens, *Colletotrichum graminicola* and *Cercospora zeina,* the causal agents of anthracnose and gray leaf spot disease, respectively (Swart *et al*., 2017). Abiotic stress experiments included roots exposed to drought (Opitz *et al*., 2014), leaves from plants challenged with salt stress, and whole above‐ground tissues exposed to temperature stress (Makarevitch *et al*., 2015) (Data S1). Between 77.8 and 98.4% of the reads per sample mapped to the AGPv4 B73 genome (Jiao *et al*., 2017). After removing lowly expressed genes (defined as fragment per kilobase of transcript per million mapped reads (FPKM) < 1 in all samples) and conducting log_2_ transformation, Pearson's correlation coefficients (PCC) of all biological replicates were greater than 0.92, except for correlations with the first replicate in the *C. graminicola* experiment. Once this replicate was removed, the average PCC value among biological replicates was 0.98 (Figure S1). The high read mapping rate and PCC values for biological replicates demonstrate the quality and reproducibility of the data set.

**Figure 1:**
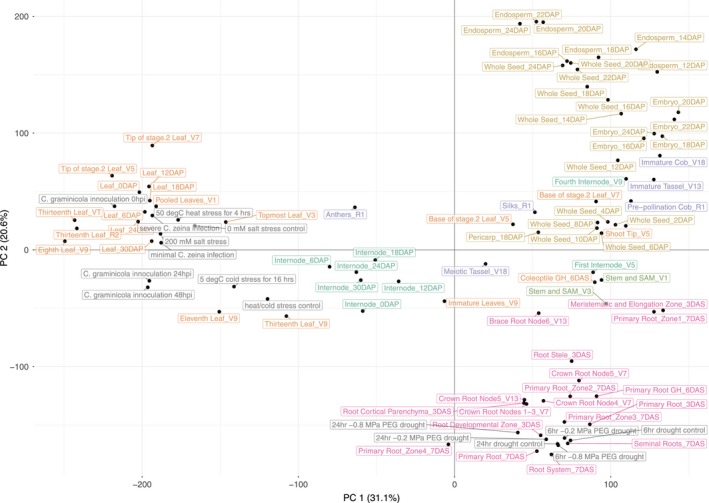
Principal component analysis of transcriptomic data. Principal component analysis was performed with the averaged biological replicate log_2_ transformed fragments per kilobase of exon model per million mapped reads for both the developmental and stress gene atlas. Samples are plotted according to their distribution on the first two principal components (PC1 and PC2), which together account for 51.7% of sample variance, and are colored according to organ. The leaf, seed, root, internode, reproductive, and shoot apical meristem organs are colored orange, yellow, pink, turquoise, purple, and green‐yellow, respectively. Samples from the stress experiments are colored gray.

To assess the relationship of the transcriptome across the samples, Pearson's correlations between all samples were hierarchically clustered and principal component analysis (PCA) was performed. In the hierarchical clustering, all six major plant organs clustered together, with separate clades for leaf, seed, root, reproductive, internode, and shoot apical meristem (SAM) organs (Figure S2, Data S2). Within each organ, samples clustered by tissue morphology, physiology, and developmental attributes. After averaging FPKM values across biological replicates and performing PCA, the first principal component (PC1) (31.1% of variance explained) and the second principal component (PC2) (20.6% of variance explained) separated the tissues by degree of tissue differentiation and development, respectively (Figure 1). The organ hierarchical clustering and principal component separation by tissue differentiation and development further indicate that the data are high quality as it separates according to the expected biological identities.

In total, 36 207 genes had an FPKM greater than zero in at least one developmental or stress gene atlas sample and 29 413 genes had an FPKM > 1, representing 92.8 and 75.4% of the annotated genes, respectively. Crown root node 5 had the largest number of genes with an FPKM > 1 (22 776 genes; 58.4%) while the endosperm 24 days after pollination had the smallest number of genes with an FPKM > 1 (14 602 genes; 37.4%) consistent with previous findings (Stelpflug *et al*., 2016). The number of genes expressed in each tissue followed the continuum observed in PCA whereby mature, differentiated tissues had the lowest number of genes expressed and immature, undifferentiated tissues had the highest number of genes expressed (Data S1). Of the 9591 genes with an FPKM ≤ 1, 33.7% were annotated as hypothetical proteins or of unknown function.

### Organ‐specific and stress‐related differentially expressed genes

**Figure 2:**
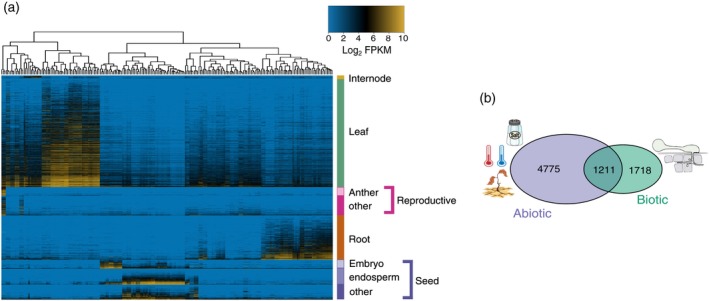
Organ‐specific and stress‐induced differential gene expression. (a) Heatmap of log_2_ fragments per kilobase of transcript per million mapped reads for the 4154 genes identified as organ‐specific in one of the six organs. Hierarchical clustering was performed on the samples and the dendrogram is on top of the heatmap. Organ and tissue‐specific genes are denoted on the right‐hand side and are grouped by organ. (b) Venn diagram of genes differentially expressed under abiotic stress (salt stress, drought (Opitz *et al*., 2014), and temperature stress (Makarevitch *et al*., 2015)), biotic stress [*Colletotrichum graminicola* and *Cercospora zeina* infection (Swart *et al*., 2017)], and both stress types.

Organ‐specific gene expression provides an opportunity to understand the fundamental questions of development as well as the identification of promoters specific to a single organ. Using the 79‐tissue developmental gene atlas data, 4154 leaf, reproductive, root, seed, or internode organ‐specific genes were identified (Data S3). Nearly 50% of the organ‐specific genes (1956) had leaf‐specific gene expression, while 19.2% (796) and 20.2% (839) of the genes had root‐ and seed‐specific gene expression, respectively (Figure 2a), whereas both the reproductive and internode organs had the lowest number of organ‐specific genes (12.3 and 1.2% respectively), consistent with previous analyses (Sekhon *et al*., 2011). Gene ontology (GO) term enrichment analysis identified top GO classes of ‘photosynthesis’ (GO:0015979) for leaf‐specific genes and ‘plant‐type cell wall organization’ (GO:0009664) for root‐specific genes (Data S4) indicating that leaf‐specific and root‐specific genes serve roles in carbon fixation and cell expansion, respectively. Seed‐specific, reproductive‐specific, and internode‐specific genes were enriched for ‘nutrient reservoir activity’ (GO:0045735), ‘protein kinase activity’ (GO:0004672), and ‘regulation of gene expression’ (GO:0010468), respectively. The seed and reproductive organ‐specific genes were further divided into tissue‐specific expression, specifically the embryo, endosperm, anther, meiotic tassel, and silk‐specific expression (Figure 2a, Data S5, Data S6). Of the seed‐specific genes, 62.7% could be classified as either endosperm‐specific or embryo‐specific, with the endosperm‐specific genes enriched for nutrient reservoir activity (GO:0045735). Of the reproductive‐specific genes, 160 genes could be classified as tissue‐specific, with 96.3% being anther‐specific and enriched for protein kinase activity (GO:0004672). This catalog of organ‐specific genes provides a resource to better understand organ development in maize and to selectively engineer maize through modification of organ‐specific genes and/or use of organ‐specific promoters.

To validate our organ‐specific genes, we utilized tissue‐specific and subcellular gene localization data available from the Maize Cell Genomics Database (Krishnakumar *et al*., 2015). In total, 14 genes in the Maize Cell Genomics Database were annotated as organ‐specific in our analyses, nine of which had confocal image evidence for stably expressed fluorescent tags. Six of the nine genes had fluorescence in the specific organ identified through our differential gene expression analyses, while the other three genes (Zm00001d022192, Zm00001d050032 and Zm00001d049099) had fluorescence in leaves rather than the reproductive or seed organs. While our expression data for these three genes indicates that they are expressed in the leaf, their expression in the reproductive or seed organs was more than two‐fold higher compared with the leaf and thus were classified as reproductive or seed‐specific rather than leaf‐specific in our study. The concordance of the organ‐specific genes identified through our analyses with the Maize Cell Genomics Database (Krishnakumar *et al*., 2015) confirms the quality of our organ‐specific gene expression data.

The developmental gene atlas reflects major transcriptional profiles involved in development, from seed to seed, with limited exposure of the plants to abiotic or biotic stress. Using RNA‐seq datasets from abiotic and biotic stress treatments, we identified DE genes following stress to improve our understanding of stress responses in B73 and to provide additional functional annotation to the AGPv4 annotated gene set. Using biotic stress experiments with *C. graminicola* and *C. zeina* (Swart *et al*., 2017) infection and three abiotic stressors, temperature (Makarevitch *et al*., 2015), salt, and drought (Opitz *et al*., 2014) (Data S1), 7704 DE genes were identified (Data S7, Figure S3). Over 60% of the genes (4775) were responsive to abiotic stress (Figure 2b), yet only 23% of the genes (1718) were responsive to biotic stress. However, 1211 genes were responsive to both biotic and abiotic stress and GO enrichment analysis identified ‘oxidation−reduction process’ (GO:0055114) and ‘DNA replication’ (GO:0006260) as the top GO terms (Data S4). Both abiotic and biotic stress had DE genes enriched for terms related to aromatic compound metabolism (GO:1901362, GO:0006558) suggesting regulation of genes encoding aromatic compound metabolism is a conserved stress response (Hildebrandt *et al*., 2015). Abiotic stress‐related DE genes were also enriched for ‘response to hormone’ (GO:0009725) while biotic stress‐related DE genes were enriched for ‘response to biotic stimulus’ (GO:0009607). These DE genes provided further functional data for the B73 genome and further understanding of the molecular mechanisms of stress responses.

### Weighted gene co‐expression network analysis

**Figure 3:**
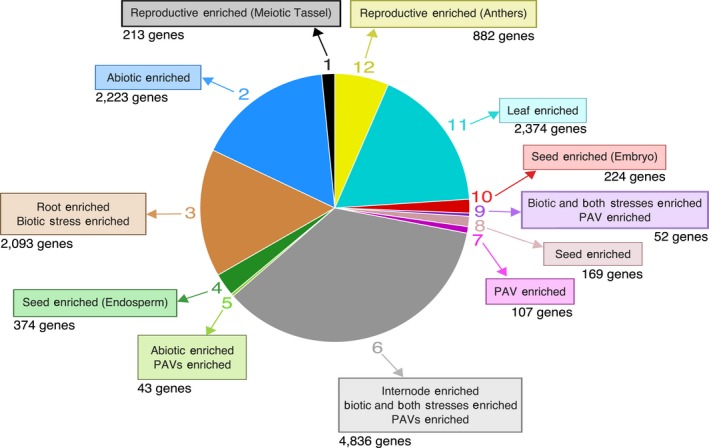
Weighted gene co‐expression network analysis modules. Pie chart of the 12 co‐expression modules identified through weighted gene co‐expression network analysis. Numbers around the outside of the pie chart indicate the module and numbers beneath the boxes indicate the number of genes present in each module. Boxes around the pie chart indicate organ‐specific, stress‐related, and presence−absence variant (Brohammer *et al*., 2018) enrichment within the module. Tissues in parentheses after an organ indicate enrichment of those tissue‐specific genes. ‘Both stresses’ refers to genes that are differentially expressed under both abiotic and biotic stress types.

Gene co‐expression analyses can reveal co‐regulated genes, pathways, and biological processes; this guilt‐by‐association method has proven fruitful for assigning function to genes with unknown function (for review see Usadel *et al*., 2009). Weighted gene co‐expression network analysis (WGCNA; Langfelder and Horvath, 2008) was performed on filtered log_2_ transformed FPKM values obtaining 12 gene modules containing 13 590 genes (Data S8). When performing hierarchical branch cutting to define modules, Module 6 was assigned to genes that did not cluster at the specified threshold and consequently is the largest module with 4836 genes (Figure S4). Enrichment analyses of organ‐specific and stress‐related DE genes within each module identified modules associated with specific organs and stress functions (Figure 3; Table S2). Module 11 is enriched for leaf‐specific genes (*P *<* *2e‐15) and GO term enrichment confirmed Module 11 is enriched for genes associated with ‘photosynthesis’ (GO:0015979) (Data S4). Reproductive‐specific genes were enriched in Modules 1 and 12 (*P *<* *2e‐15) corresponding to GO enrichment terms of ‘sexual reproduction’ (GO:0019953). Specifically, meiotic tassel and anther‐specific genes were enriched in Modules 1 and 12, respectively (*P *<* *2e‐15). Three modules (Modules 4, 8, and 10) are enriched for seed‐specific genes (*P *<* *2e‐15) with GO term enrichment classes of ‘nutrient reservoir activity’ (GO:0045735) and ‘embryo development’ (GO:0009790). Module 4 is enriched for endosperm‐specific genes and Module 10 is enriched for embryo‐specific genes (*P *<* *2e‐15). Module 3 is enriched for root‐specific and biotic‐related DE genes (*P *<* *0.001) and GO enrichment identified ‘response to oxidative stress’ (GO:0006979) and ‘cell wall organization or biogenesis’ (GO:0071554). Module 6 was jointly enriched for internode‐specific genes, biotic‐related DE genes, and DE genes under both biotic and abiotic stress (*P *<* *5e‐9). The enrichment of organ‐specific and stress DE genes in Module 6 suggests that there may be co‐expressed groups of genes within this module that did not reach the branch cutting threshold and application of a more stringent threshold may separate these into separate modules. Two modules (Modules 2 and 5) were enriched for abiotic‐related DE genes (*P *<* *2e‐15) and Module 9 was enriched for biotic‐related DE genes and DE genes under both biotic and abiotic stress (*P *<* *4e‐5). Among the modules enriched for stress DE genes, Module 2 had GO enrichment for core functions such as ‘DNA replication’ (GO:0006260) and ‘primary metabolic process’ (GO:0044238).

Gene and module connectivity measures are important indicators of central pathway genes and module cohesiveness. The WGCNA R package (Langfelder and Horvath, 2008) outputs a total connectivity measure (kTotal) which sums all row values for the given gene in the transformed correlation matrix (i.e., the adjacency matrix). Similarly, there is an intra‐modular connectivity measure (kWithin) in which row values are summed only for the genes within the same module. The average kTotal and kWithin values were 177.96 (±139.73) and 138.88 (±137.21) (Table 1). The modules with the most genes had the highest average kTotal with the exception of Module 6 indicating that highly connected hub genes tend to be in larger modules enriched for core functions. Module cohesiveness can be assessed by evaluating the difference between intra‐modular connectivity and inter‐modular connectivity (kDiff). A module with a positive average kDiff suggests that the genes in the module have more connections to each other than to genes outside the module. Most modules had positive kDiff averages (Table 1), though Modules 8 and 10 had lower averages of 5.31 and 4.46, respectively. Modules 4, 8, and 10 are seed‐specific modules, with Modules 4 and 10 enriched for endosperm‐specific and embryo‐specific genes, respectively, while Module 8 is primarily expressed in the whole seed (Figure S5). The low kDiff average is likely due to the overlapping functions of these three modules in seed development leading to high inter‐modular connectivity between the modules. Module 6 unsurprisingly had a slightly negative kDiff of −0.41 and two other modules (5 and 9) had negative kDiff averages of −1.73 and −18.87, respectively suggesting that Modules 5 and 9 may not be individual co‐expression modules and the branch cutting threshold could have been slightly adjusted to prevent their identification. These co‐expression modules and the gene connections provide an extensive resource for understanding co‐regulated genes and pathways.

**Table 1:** Weighted gene co‐expression network analysis module connectivity

Module	kTotal	kWithin	kDiff	kDiff/kTotal
1	93.90	80.48	67.06	0.71
2	209.20	180.47	151.75	0.73
3	260.74	197.33	133.93	0.51
4	97.91	73.84	49.78	0.51
5	27.02	12.65	−1.73	−0.06
6	92.28	45.94	−0.41	0.00
7	42.53	34.56	26.59	0.63
8	70.05	37.68	5.31	0.08
9	50.46	15.79	−18.87	−0.37
10	67.88	36.17	4.46	0.07
11	230.05	199.98	169.91	0.74
12	366.60	353.71	340.82	0.93

Average total (kTotal) and intramodular (kWithin) connectivity values for each co‐expression module, with the difference in connectivity between intramodular and intermodular connectivity (kDiff) provided. The ratio between the kDiff and kTotal is also provided, with values ranging from −1 to 1. A 1 indicates no genes are connected to other modules and a −1 means no genes have connections within the module.

### Understanding dispensable gene function through expression analyses

**Figure 4:**
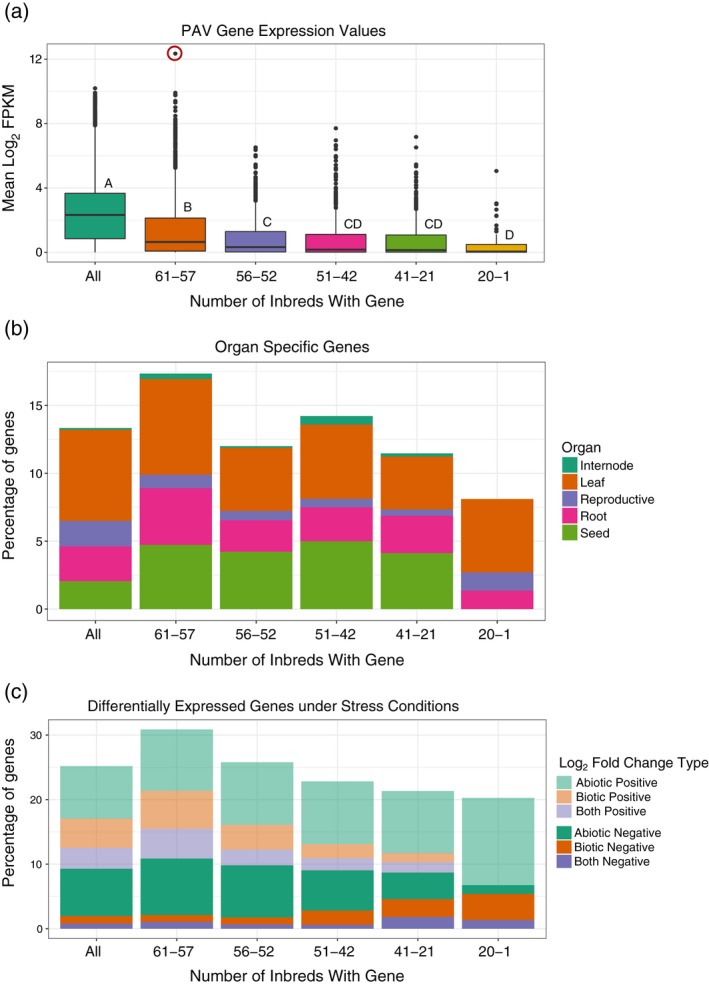
Presence−absence variant differential expression enrichment. (a) Box plot of log_2_ fragments per kilobase of exon model per million mapped reads from the developmental gene atlas for genes classified by how many inbreds contain the gene. Genes were classified into the subcategories using the presence−absence variant data from Brohammer *et al*. (2018), with each color corresponding to a subcategory. Letters refer to significant differences from Tukey's test using a *P*‐value threshold of 0.05. Zm00001d031168 is circled in red. (b) Bar plot of the percentage of genes in each presence−absence variant category which are organ‐specific. The percentages are further subdivided by organ type. (c) Bar plot of the percentage of genes in each presence−absence variant category which are differentially expressed under stress. The stress types are further subdivided into abiotic stress [salt stress, drought (Opitz *et al*., 2014), and temperature stress (Makarevitch *et al*., 2015)], biotic stress (*Colletotrichum graminicola* and *Cercospora zeina* infection (Swart *et al*., 2017)), and both stress types. The shade of color indicates if the gene in each stress type has a positive or negative log_2_ fold change.

Hirsch *et al*. (2016) identified PAVs from a comparison of the B73 (AGPv3) and PH207 draft assemblies, and found these to be more lowly expressed and organ‐specific compared with core genes (Hirsch *et al*., 2016). Brohammer *et al*. (2018) identified 11 971 PAVs from the anchored AGPv4 B73 loci using resequencing data from 62 inbreds and defining a gene as a PAV if the CDS coverage was less than 20% (Brohammer *et al*., 2018). Of the PAVs identified in the Brohammer *et al*. (2018) study, 43.1% were present in 61–57 inbreds (broadly dispersed) while 4.9% were present in 20–1 inbreds (restricted dispersion) (Table S1). Using our set of 274 diverse developmental and stress transcriptome samples, 4420 PAVs were expressed with an FPKM > 1 accounting for 15.0% of the expressed genes in B73. Relative to core genes present in all inbreds, PAVs had lower mean expression abundances within the 79 developmental gene atlas samples which were inversely correlated with the frequency of the PAV in the panel (Figure 4a). Zm00001d031168, an ortholog of the *Arabidopsis thaliana* cold, circadian rhythm, and RNA binding 2 gene (AT2G21660), had a mean log_2_ FPKM expression of 12.4 and was a PAV with 61–57 inbreds encoding Zm00001d031168. Interestingly, among all expressed genes, genes with organ‐specific and stress‐related gene expression were enriched for PAVs (*P *<* *0.002) with PAVs present in 61–57 inbreds being the main contributor of PAV enrichment among organ‐specific and stress‐related DE genes (Figure 4b,c). Seed‐specific genes were enriched among PAVs present in 61–42 inbreds (*P *<* *0.008) with endosperm‐specific genes enriched among PAVs present in 61–57 inbreds and 51–42 inbreds (*P *<* *6e‐11). Seed‐specific PAVs were enriched for ‘defense responses’ (GO:0006952). Upregulated abiotic stress‐induced DE genes were enriched among PAVs present in 51–21 inbreds (*P *<* *0.04) and enriched for ‘response to hormone’ (GO:0009725). Our results are consistent with previous findings of low PAV expression and increased PAV organ‐specificity (Hirsch *et al*., 2016) and expand the role of PAVs to include response to abiotic and biotic stress. Additionally, the lower expression values and lack of functional enrichment among PAVs present in a small number of inbreds indicates that this subset of PAVs may be undergoing pseudogenization (Tan *et al*., 2012) or have highly specific expression patterns and functions not captured in these studies.

**Figure 5:**
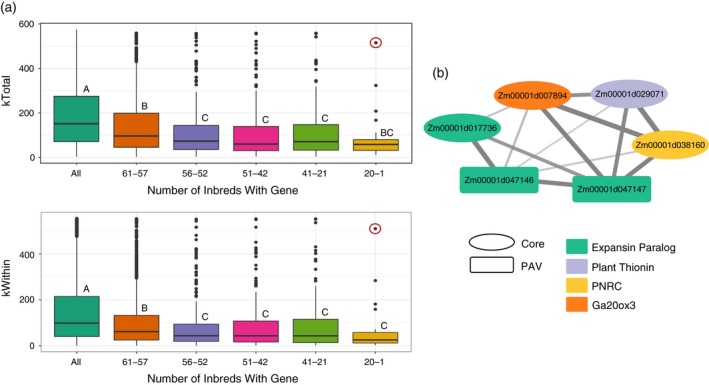
Presence−absence variant connectivity in weighted gene co‐expression network analysis. (a) Box plot of the total connectivity measure (kTotal) and the intramodular connectivity measure (kWithin) for each gene present in a co‐expression module categorized by how many inbreds contain the gene. Genes were classified into the sub‐categories using the presence−absence variant data from Brohammer *et al*. (2018), with each color corresponding to a subcategory. Letters refer to significant differences from Tukey's test with a *P*‐value threshold of 0.05. The gene circled in red is Zm00001d047146 (expansin β1). (b) Network of genes to which Zm00001d047146 is connected that are compensated by two other expansin paralogs. The weighted gene network co‐expression analysis identified a co‐expression network in which the nodes are the genes and the edges are the gene connections. The thickness of the edge indicates the weight of the connection and the shape of the gene indicates gene type (a core gene present in all inbreds or a presence−absence variant). The expansin paralogs are colored turquoise, plant thionin is colored purple, proline‐rich nuclear receptor coactivator (PRNC) is colored yellow, and gibberellin 20 oxidase 3 (GA20ox3) is colored orange.

In the WGCNA, Modules 5, 6, 7, and 9 were enriched for PAVs (*P *<* *6e‐6) and Module 7 was also enriched for stress‐related DE genes (Figure 3; Table S2). These modules had negative average kDiff values (Table 1) suggesting that PAVs associated with stress responses interact with pathways that are not co‐expressed. Furthermore, when comparing core gene and PAV network connectivity, PAVs had significantly lower total and intra‐modular connectivity, with smaller connectivity values as fewer inbreds encoded the gene (Figure 5a). This pattern is consistent with that observed for PAV expression and further suggests that PAVs have discrete expression patterns that are not captured under the conditions sampled in this study or are on a path to pseudogenization.

Interestingly, 52 PAVs did not follow this pattern and had a kTotal value greater than 500 (top 4% of genes), of which 42 were from Module 12 and enriched for ‘sexual reproduction’ (GO:0019953) (Data S4, Data S9). In total, 66.7% of the 52 outlier PAVs were present in 61–57 inbreds and one gene (Zm00001d047146) was missing in 49 inbreds. Zm00001d047146 is a classical gene annotated as expansin β1 (EXPβ1; http://www.maizegdb.org/), a protein shown to be involved in pollen tube penetration of silk tissue (Valdivia *et al*., 2009; Tabuchi *et al*., 2011). EXPβ1 has 543 gene connections in the co‐expression network (Data S9) and is part of a monocot‐specific orthologous group in which EXPβ1 has 14 other paralogs in B73. Among the paralogs, eight are PAVs, of which, two are classical genes: EXPβ1 (Zm00001d047146) and EXPβ10C (Zm00001d040609). EXPβ1 is connected to 13 of the 14 other EXPβ1 paralogs and the overlapping gene connections among the paralogs was assessed to investigate the degree of compensation for EXPβ1 loss. Of the 543 genes connected to EXPβ1, 466 genes were connected to all 14 paralogs with just three genes connected to less than five of the paralogs. Based on sequence similarity to *A. thaliana* and *O. sativa* genes, a plant thionin (Zm00001d029071) and a proline‐rich nuclear receptor coactivator (Zm00001d038160) were connected to EXPβ1 and the PAV paralog Zm00001d047147 (Figure 5b). Plant thionins are antimicrobial peptides (Tam *et al*., 2015) and proteins with proline‐rich receptor coactivator motifs have been shown to be involved in mRNA de‐capping (Wurm *et al*., 2016). The third gene (Zm00001d007894), which has sequence similarity to gibberellin 20 oxidase 3 (GA20ox3) was connected to two other paralogs (Zm00001d047147 and Zm00001d017736). GA20ox3 functions in gibberellin biosynthesis and has been shown to confer enhanced resistance against biotic stress in rice (Qin *et al*., 2012). While Zm00001d017736 is a core gene and could replace EXPβ1 and the other PAV paralog, it has a weaker connectivity weight suggesting that this connection is not as important (Figure 5b). All three of these genes are putatively involved in plant defense responses and were the only genes not strongly compensated for by another non‐PAV EXPβ1 paralog suggesting that the loss of this gene may be associated with an altered stress response rather than an altered reproductive capacity. Indeed, expansins have been shown to be involved in plant stress responses (Marowa *et al*., 2016). This example demonstrates the utility of co‐expression analyses in connecting gene function and pathway information to determine potential genetic mechanisms of plant phenotype.

Among non‐expansin genes in the 52 outlier PAVs, 88.6% (39) did not have a paralog(s) with overlapping gene connections in the co‐expression network. Furthermore, 31 of these 39 non‐expansin outlier PAVs are single copy in the B73 genome; of these 13 are annotated with functional annotation corresponding to involvement in core processes such as nuclear transport, protein phosphorylation, and vesicle trafficking while the other 18 are annotated as ‘hypothetical’ or ‘conserved hypothetical’. It is unknown how pathways are affected when highly connected PAVs that lack a paralog which can compensate for lost connections are absent from an inbred. Future studies focused on these singleton PAVs would be informative to understand network changes and how these relate to differences in biological processes.

### Website and eFP browser development for open‐source data dissemination

**Figure 6:**
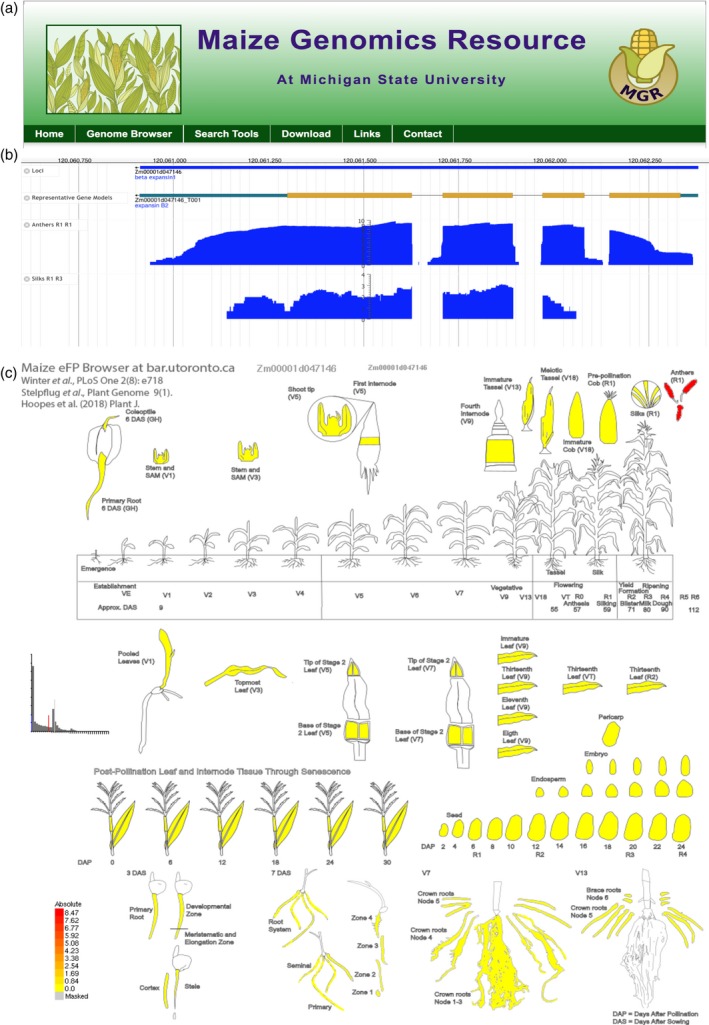
Maize genomics resource and eFP browser. (a) Header for the Maize Genomics Resource website, which provides search and query tools for quick access to the data via a BLAST server, genome browser, and gene report pages. (b) JBrowse view of Zm00001d047146 showing the locus, representative transcript, anther tissue *XY* plot, and silk *XY* plot. (c) The Bio‐Analytic Resource for Plant Biology maize eFP browser for the developmental gene atlas expression data. Zm00001d047146 expression abundances are shown, with red indicating the highest absolute expression of the gene.

The extensive AGPv4 transcriptome resource generated here provides a multi‐faceted and comprehensive database for improved functional annotation and we have deployed a robust set of on‐line tools to data‐mine these transcriptome datasets. We created the MGR website (http://maize.plantbiology.msu.edu) to provide search and query tools for quick access to the data presented here via a BLAST server, a genome browser, and gene report pages in a layout similar to the Rice Genome Annotation Project and Spud DB (Ouyang *et al*., 2007; Kawahara *et al*., 2013; Hirsch *et al*., 2014b) (Figure 6a). The MGR genome browser, an instance of JBrowse (Buels *et al*., 2016) with 561 tracks contains the AGPv4 assembly and annotation (Jiao *et al*., 2017) along with RNA‐seq coverage wiggle and XY plots from the libraries analyzed here (Figure 6b). Additional tracks such as HapMap v3.2.1 variants (Bukowski *et al*., 2018), RNA‐seq variants (Hirsch *et al*., 2014a; Diepenbrock *et al*., 2017), best protein matches from select species in the Poaceae family, and transposable element annotation provide additional contextual information for gene function interpretation. Functional annotations including Pfam, InterPro and GO annotation were generated for the AGPv4 gene models and are searchable by keyword and accession with link outs to Gene Reports for the matching gene models. A Gene Report page contains the gene model identifier, alternative isoforms, gene model attributes, genome browser view, gene model/CDS/peptide sequences, associated GO annotations, Pfam hits, InterPro hits, orthologous group membership, expression profiles (FPKM, DE gene expression), WGCNA modules, and connectivity values. Within the MGR, a sequence retrieval tool for the AGPv4 genome, gene models, and surrounding gene model regions are provided. A BLAST server is also available to allow users to search *Z. mays* B73, including older versions, PH207 (Hirsch *et al*., 2016), Mo17 (Sun *et al*., 2018), W22 (Springer *et al*., 2018), and other assemblies and annotation. All data sets are also available for download including the WGCNA and DE gene lists. The MGR provides an easy to use resource for the maize community that implements updated functional annotation and analyses for the AGPv4 assembly and gene annotation.

Data visualization is intuitive to the user and the Bio‐Analytic Resource for Plant Biology (BAR) (bar.utoronto.ca) provides web‐based tools for gene expression visualization. Two new views, called ‘Hoopes *et al*. Atlas’ and ‘Hoopes *et al*. Stress’ were generated for the maize eFP (‘electronic Fluorescent Pictograph’) Browser (http://bar.utoronto.ca/efp_maize) (Li *et al*., 2010) (Figure 6c). The eFP images for any gene may be explored at the BAR maize eFP Browser website, or viewed at the MGR via a web service provided by the BAR, thereby providing a rapid means of assessing where and when a gene is expressed.

## Conclusion

We have developed a comprehensive transcriptomic resource for the maize community to better interpret and utilize genomic, transcriptomic, genetic, and functional datasets with the long‐read‐based B73 AGPv4 genome assembly and annotation. The unified web interface provides search and query tools for easy data access, and gene expression visualization is available with the eFP Browser. Access to a 79 tissue, 6‐organ developmental gene atlas coupled with a set of five abiotic/biotic stress transcriptome datasets permitted robust annotation of the 39 005 genes within the AGPv4 annotated gene set including identification of 4154 organ‐specific genes and 7704 genes that are differentially expressed following stress treatment, and 12 co‐expression modules. The utility of a robust, comprehensive transcriptome resource permitted further characterization of PAVs within a 62‐member diversity panel revealing that not only are PAVs lowly expressed, but also that their expression is correlated with their dispersion frequency across accessions. Interestingly, seed‐specific and stress‐induced DE genes are enriched for PAVs and co‐expression modules enriched for PAVs also tend to be enriched for stress‐induced DE genes. Together, these results suggest PAVs are associated with environmental adaptation responses and demonstrate the utility of the resource in elucidating gene function.

## Experimental Procedures

### Mapping transcriptome reads and calling gene expression values

FASTQ files from the National Center for Biotechnology Information (NCBI) (https://www.ncbi.nlm.nih.gov/) (Data S1) were downloaded and read quality was assessed with FastQC (v0.11.5) (https://www.bioinformatics.babraham.ac.uk/projects/fastqc/) and MultiQC (v1.0) (Ewels *et al*., 2016). Adapters, low quality bases (Q < 20), and undefined bases at the ends of reads were removed using Cutadapt (v1.12) (Martin, 2011), retaining reads ≥ 30 nt in length. The cleaned paired‐end reads were then concatenated into a single file. All cleaned reads were aligned to the *Z. mays* B73 AGPv4 genome assembly (Jiao *et al*., 2017) with Bowtie2 software (v2.2.3) (Langmead and Salzberg, 2012) and TopHat2 (v2.0.14) (Kim *et al*., 2013) using the unstranded mode and a maximum intron length of 60 kb. Expression abundances (FPKM) for *Z. mays* B73 AGPv4 genes (Jiao *et al*., 2017) were quantified with Cufflinks (v2.2.1) (Trapnell *et al*., 2010) in unstranded mode, a maximum intron length of 60 kb, and using reference bias correction.

### Quality assessment of transcriptomic data

Genes with FPKM values > 1 in at least one sample were log_2_ transformed and PCCs were calculated for all samples including biological replicates using the R ‘cor’ function. One biological replicate in the *C. graminicola* experiment with a PCC value below 0.92 was removed. PCC values across all samples were hierarchically clustered and visualized in a heatmap using the R package ‘gplots’ (Alexa and Rahnenfuhrer, 2016) ‘heatmap.2’ function. PCA was conducted using the log_2_ transformed FPKM values of expressed genes averaged by biological replicate and the R function ‘prcomp’. The R package ‘factoextra’ (Kassambara and Mundt, 2017) was then used to determine the extent of variance explained by each component using the ‘get_eigenvalue’ function and to extract the sample coordinates using the ‘get_pca_ind’ function. The PCA was visualized via the R packages ‘ggplot2’ (Wickham, 2009) and ‘RColorBrewer’ (Neuwirth, 2014). To assess differences in gene expression levels, Tukey's test was performed after normalizing the data in R and a *P*‐value of 0.05 was used as the significance threshold.

### Enrichment analyses

Locus GO terms were assigned by searching the AGPv4 proteome using InterProScan (v5.14.53.0) (Jones *et al*., 2014) with the ‘goterms’ option. For all GO term enrichment analyses, the Bioconductor R package ‘topGO’ (Alexa and Rahnenfuhrer, 2016) was used to perform a Fisher's exact test with the classic method using the custom GO term assignments to assign a *P*‐value to the GO term classes. The *P*‐values were then adjusted using the false discovery rate (FDR) correction and GO terms with an adjusted *P *<* *0.05 were retained. All other enrichment analyses for the presence of PAVs, organ‐specific genes, and stress‐related DE genes were conducted via the chi‐squared test in R using ‘chisq.test’ function and the *P*‐values were adjusted using the Bonferroni correction.

### Differential gene expression analyses

Read counts per gene were obtained from the RNA‐seq alignments using HTSeq (v0.9.1) (Anders *et al*., 2015) in unstranded union mode. Differentially expressed genes were identified from the gene read counts using DESeq2 (Love *et al*., 2014) with an alpha level of 0.01. For organ‐specific gene expression, an organ was defined similarly to Sekhon *et al*. (2011) with some modifications (Sekhon *et al*., 2011). The endosperm and embryo were grouped into a single organ (seed) and the cob, silk, tassel, and anthers were grouped into one reproductive organ. The SAM was also considered a separate organ establishing six organs for analyses: internode, leaf, reproductive, root, seed, and SAM. Each organ from the developmental gene atlas was individually contrasted against the other five organs retaining genes with an adjusted *P *<* *0.01 and a log_2_ fold change >2. A gene was considered organ‐specific if it met these criteria in the organ of interest when compared against all other organs. Tissue‐specific gene expression was similarly characterized for seed and reproductive tissues. Using read counts for genes previously identified as seed‐ or reproductive‐specific, the seed and reproductive tissues were contrasted against the other tissues in its respective organ. A gene was considered tissue‐specific if it had an adjusted *P *<* *0.01 and a log_2_ fold change >2 in all tissue comparisons. For stress‐related differential expression, each treatment was contrasted against its respective experimental control, retaining genes with an adjusted *P *<* *0.01 and a log_2_ fold change <−2 or a log_2_ fold change >2. A gene was considered DE under both abiotic and biotic stress if it met these criteria in at least one treatment from both stress types. The organ‐specific heatmap was generated with the R package ‘gplots’ (Alexa and Rahnenfuhrer, 2016) ‘heatmap.2’ function and all other plots were generated with the R packages ‘ggplot2’ (Wickham, 2009) and ‘RColorBrewer’ (Neuwirth, 2014).

### Weighted gene co‐expression network analysis

Genes with an FPKM value > 5 in at least one sample were log_2_ transformed and retained only if they had a coefficient of variance > 0.6. The WGCNA R package (Langfelder and Horvath, 2008) was used with the filtered and transformed FPKM values to define modules and gene connections. Briefly, the ‘pickSoftThreshold’ function was used to determine the optimal power to transform the correlation matrix and the value of 12 provided the optimal scale free topology fit. The ‘blockwiseModules’ function was used to obtain gene modules using a cut height of 0.9 and the ‘intramodularConnectivity’ function was used to obtain connectivity measures using the adjacency matrix, which was generated using a signed network type. Cytoscape files (Shannon *et al*., 2003) were generated using the ‘exportNetworkToCytoscape’ function with an adjacency value threshold of 0.5 for calling a network edge. To assess differences in gene connectivity, Tukey's test was conducted after normalizing the data in R using a *P*‐value of 0.05 as the significance threshold.

### Maize genomics resource website development

Additional analyses were conducted to provide enhanced functional annotation of the gene models. AGPv4 protein gene model sequences were searched against the Pfam (v31.0) (Finn *et al*., 2016) database using the HMMER (v3.1b2) (Mistry *et al*., 2013) ‘hmmscan’ function. Proteins were also searched against InterPro (v67.0) (Finn *et al*., 2017) using InterProScan (v5.28.67.0) (Jones *et al*., 2014). Results from both were used to find protein domains and motifs. The ‘goterms’ option was used with InterProScan to assign GO terms to each gene model. The longest isoform for each locus was assigned as the representative isoform. The representative gene model protein sequences from *Amborella trichopoda* (v1) (Amborella Genome Project, 2013), *Arabidopsis thaliana* (TAIR10) (Lamesch *et al*., 2012), *Oryza sativa* (RGAPv7) (Ouyang *et al*., 2007), *Sorghum bicolor* (v3) (McCormick *et al*., 2018), and *Z. mays* (AGPv4) (Jiao *et al*., 2017) were run with OrthoFinder (v2.2.0) (Emms and Kelly, 2015) using default parameters to obtain orthologous and paralogous groups. Functional annotation was generated by searching the AGPv4 gene models against the *A. thaliana* proteome (TAIR10) (Lamesch *et al*., 2012), Swiss‐Prot (Bairoch and Apweiler, 2000), and Pfam (v29) (Finn *et al*., 2016). Functional annotation was assigned from the first match found in the results in the order: TAIR10, Swiss‐Prot, and Pfam. If the first match had the function ‘hypothetical’, the assigned function was ‘conserved hypothetical’. Gene models without hits in all three databases were annotated as ‘hypothetical’.

The two new views (‘Hoopes *et al*. Atlas’ and ‘Hoopes *et al*. Stress’) for the Maize eFP Browser were generated by creating appropriate images and XML files as described in the original eFP Browser paper (Winter *et al*., 2007). RNA‐seq data as FPKM values were databased on the BAR server to provide rapid access to the data by the eFP Browser engine, which ‘paints’ the expression data onto images representing the samples used to generate the RNA‐seq data. We also enabled the eFP images to be generated via a web service call such that they can be embedded in any website, such as the MGR.

## Accession numbers

Raw transcriptomic reads are available from NCBI via the following BioProject IDs: PRJNA171684, PRJEB10574, PRJNA226757, PRJNA244661, PRJNA323555, and PRJNA369690. All other data sets including the protein domain and motif matches from HMMER and InterPro, orthologous and paralogous groups from OrthoFinder, GO term assignments, and functional annotation are available from Dryad via the following DOI: https://doi.org/10.5061/dryad.5p58q34. All data sets are also available for download via the MGR download page (http://maize.plantbiology.msu.edu/MSU_func_download.shtml).

## Conflict of interest

The authors declare no conflicts of interest.

## Supporting information


**Figure S1.** Biological replicate Pearson's correlation coefficients.

**Figure S2.** Pearson's correlation coefficient heatmap.

**Figure S3.** Stress‐induced gene expression Venn diagram.

**Figure S4.** Topological overlap matrix heatmap.

**Figure S5.** *Z*‐score expression graph for Modules 4, 7, 8 and 10.

**Table S1.** Number of genes missing by inbred

**Table S2.** Weighted gene co‐expression network analysis module composition

**Data S1.** List of transcriptomic libraries analyzed.

**Data S2.** PCC matrix for tissue samples.

**Data S3.** Organ‐specific genes.

**Data S4.** GO enrichment analyses.

**Data S5.** Seed tissue‐specific genes.

**Data S6.** Reproductive tissue‐specific genes.

**Data S7.** Stress‐related differentially expressed genes.

**Data S8.** Weighted gene co‐expression network analysis modules and connectivities.

**Data S9.** PAV connectivity outliers and EXPβ1 connections.
