# Supplementary material for: An updated gene atlas for maize reveals organ‐specific and stress‐induced genes
Source: Plant J. 2019 Jan 22;97(6):1154–67. doi: 10.1111/tpj.14184 (PMC6850026; doi:10.1111/tpj.14184)
Supplement: Supplementary file 1 — Figure S1. Biological replicate Pearson's correlation coefficients. [file TPJ-97-1154-s001.pdf]

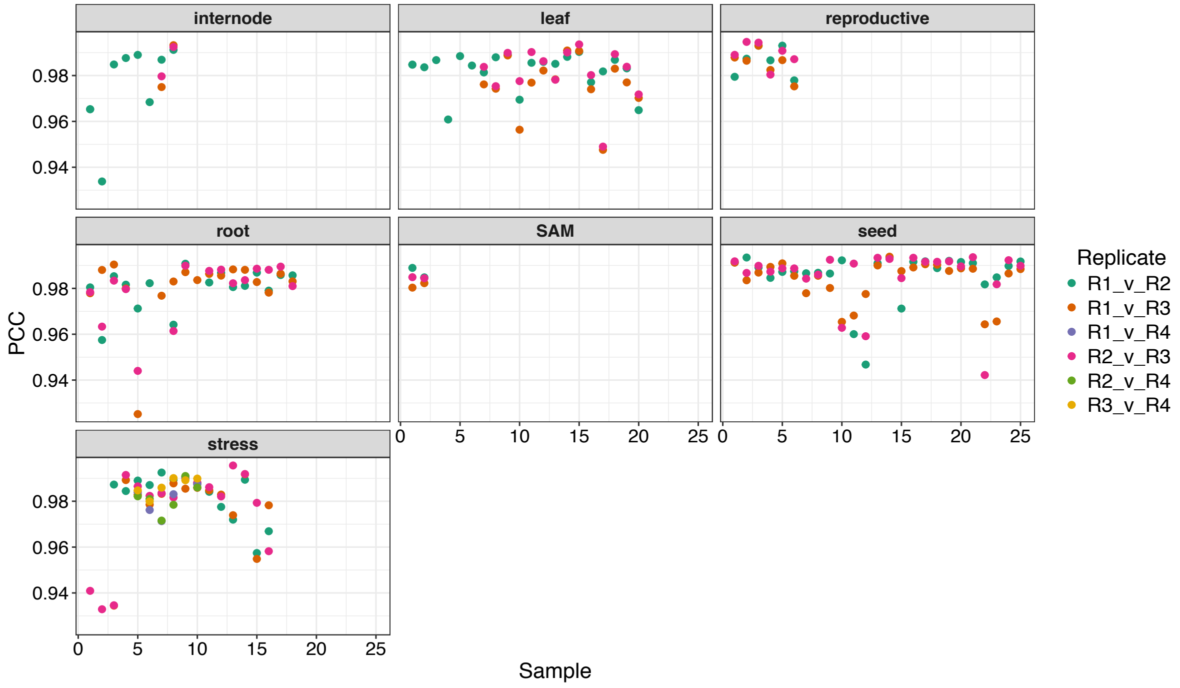

Figure S1: Biological Replicate Pearson's Correlation Coefficients
Pearson's correlation coefficients (PCC) were calculated among the biological replicates for each sample. The coefficients are plotted by sample and further separated by organ type and stress experiments. 'SAM' refers to shoot apical meristem. Coloring indicates which replicates were compared in a pair-wise fashion.
