# Supplementary material for: An updated gene atlas for maize reveals organ‐specific and stress‐induced genes
Source: Plant J. 2019 Jan 22;97(6):1154–67. doi: 10.1111/tpj.14184 (PMC6850026; doi:10.1111/tpj.14184)
Supplement: Supplementary file 2 — Figure S2. Pearson's correlation coefficient heatmap. [file TPJ-97-1154-s002.pdf]

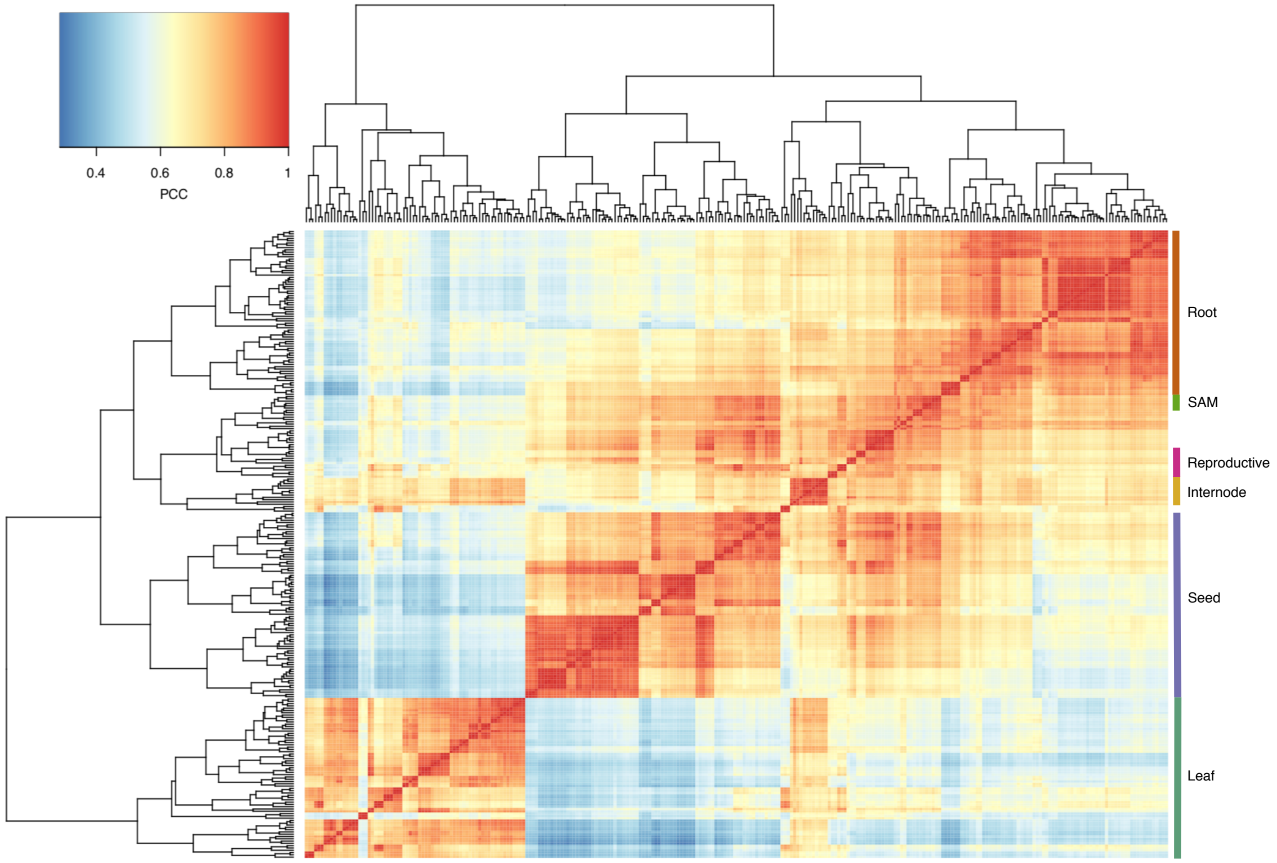

Figure S2: Pearson's Correlation Coefficient Matrix Heatmap

Pearson's Correlation Coefficients (PCC) were calculated for all pairwise comparisons of the samples and the resulting matrix was graphed in a heatmap. Hierarchical clustering of the samples was performed and the dendrograms were plotted on both axes. Samples clustering for all six organs (leaf, seed, root, reproductive, internode, and shoot apical meristem (SAM)) are indicated on the right by the vertical bars. Samples not clustering for a specific organ were left unlabeled.
