# Supplementary material for: An updated gene atlas for maize reveals organ‐specific and stress‐induced genes
Source: Plant J. 2019 Jan 22;97(6):1154–67. doi: 10.1111/tpj.14184 (PMC6850026; doi:10.1111/tpj.14184)
Supplement: Supplementary file 3 — Figure S3. Stress‐induced gene expression Venn diagram. [file TPJ-97-1154-s003.pdf]

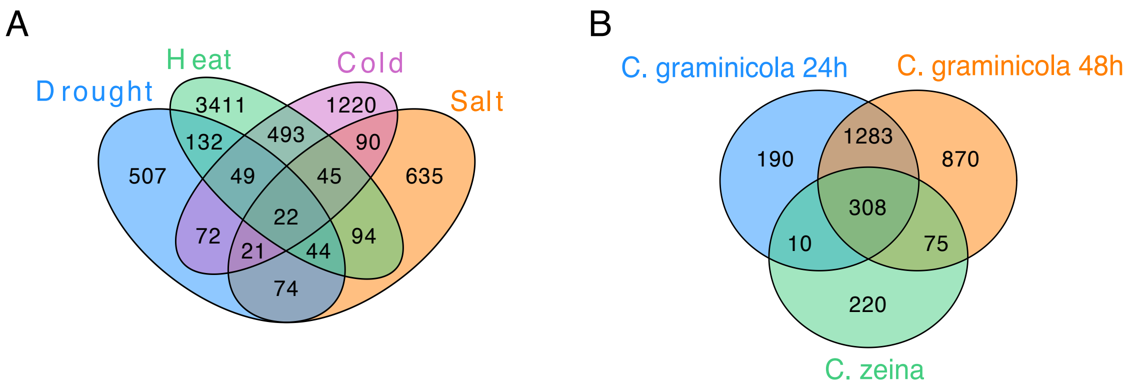

Figure S3: Stress-Induced Gene Expression Venn Diagram

A. Venn diagram of genes differentially expressed under drought, heat, cold, and salt stress. **B.** Venn diagram of genes differentially expressed under *Colletotrichum graminicola* and *Cercospora zeina* infection. *C. graminicola* infection was distinguished by two time points: 24 hours and 48 hours after infection.
