# Supplementary material for: An updated gene atlas for maize reveals organ‐specific and stress‐induced genes
Source: Plant J. 2019 Jan 22;97(6):1154–67. doi: 10.1111/tpj.14184 (PMC6850026; doi:10.1111/tpj.14184)
Supplement: Supplementary file 4 — Figure S4. Topological overlap matrix heatmap. [file TPJ-97-1154-s004.pdf]

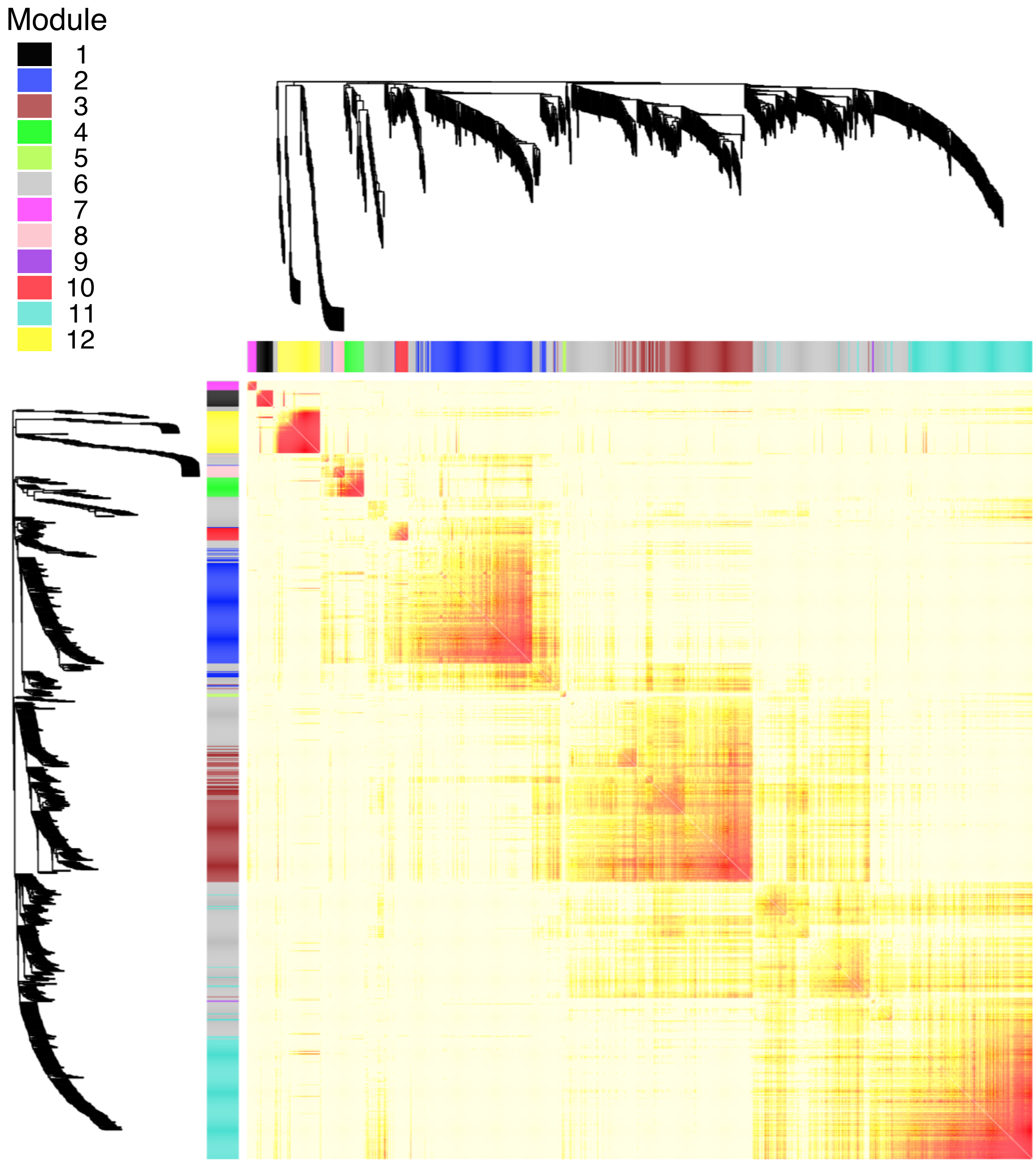

Figure S4: Topological Overlap Matrix Heatmap

During weighted gene co-expression network analysis, the topological overlap similarity measure between genes is calculated to determine module membership. The topological overlap matrix values for 1,000 randomly selected genes in the analysis were graphed in a heatmap with red indicating high interconnectedness. Hierarchical clustering is performed on the matrix and tree cutting is used to define co-expression modules. The trees on both axes represent clustering genes with the color beneath the tree indicating which Module each gene belongs to.
