# Supplementary material for: An updated gene atlas for maize reveals organ‐specific and stress‐induced genes
Source: Plant J. 2019 Jan 22;97(6):1154–67. doi: 10.1111/tpj.14184 (PMC6850026; doi:10.1111/tpj.14184)
Supplement: Supplementary file 5 — Figure S5. Z‐score expression graph for Modules 4, 7, 8 and 10. [file TPJ-97-1154-s005.pdf]

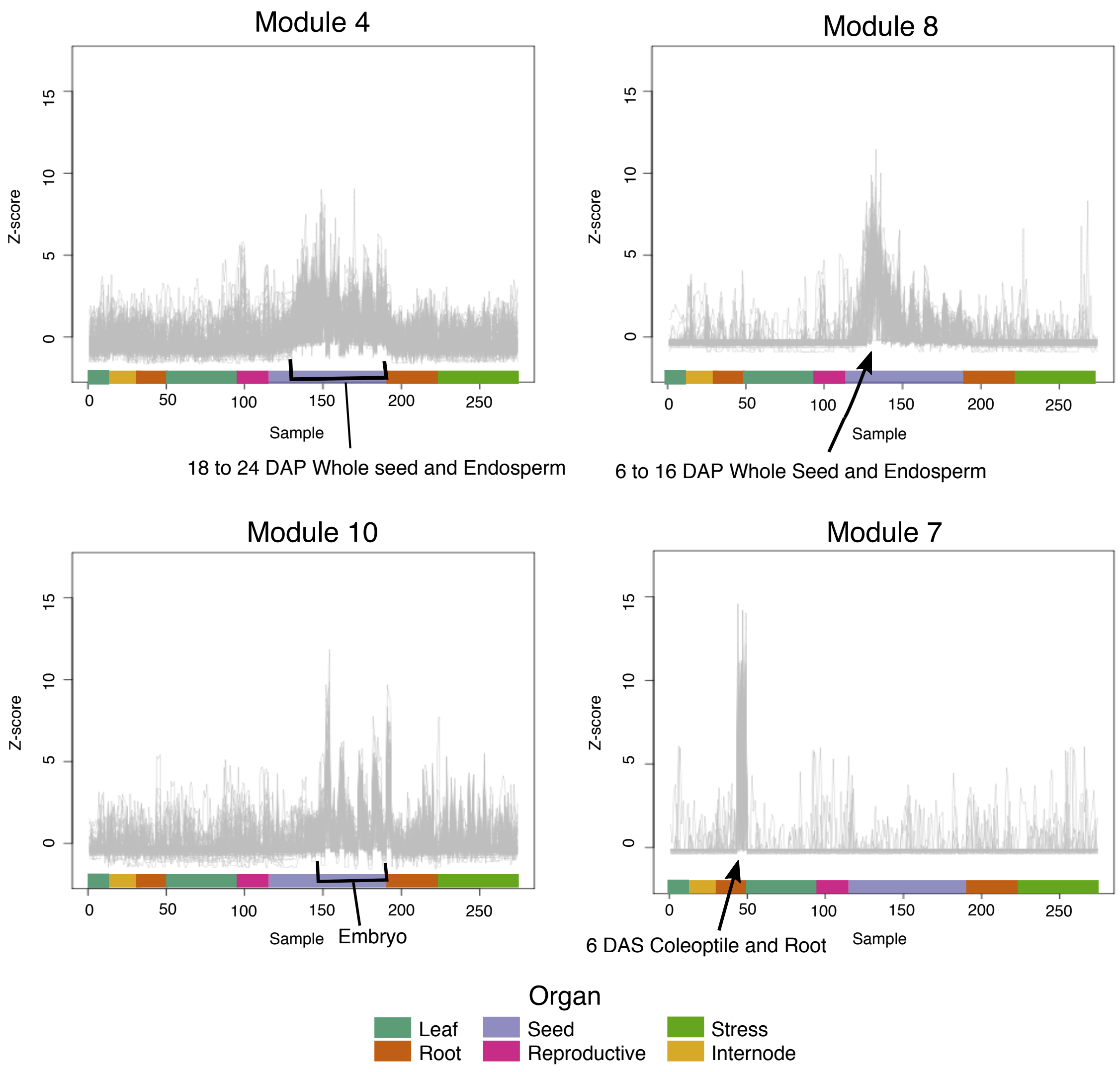

Figure S5: Z Score Expression Graph for Modules 4, 7, 8, and 10

Z score expression values were calculated from the Fragments Per Kilobase of transcript per Million mapped reads for each co-expression module identified from weighted gene co-expression network analysis. Z scores were graphed by sample for all genes in Modules 4, 7, 8, and 10. Organs and samples with peak expression values in each module are indicated on the x-axis with the horizontal bars and text, respectively. 'DAP' refers to days after pollination and 'DAS' refers to days after sowing.
