# Supplementary material for: An updated gene atlas for maize reveals organ‐specific and stress‐induced genes
Source: Plant J. 2019 Jan 22;97(6):1154–67. doi: 10.1111/tpj.14184 (PMC6850026; doi:10.1111/tpj.14184)
Supplement: Supplementary file 6 — Table S1. Number of genes missing by inbred [file TPJ-97-1154-s006.pdf]

Table S1: Number of Genes Present By Inbred

Number of Inbreds	All	61-57	56-52	51-42	41-21	20-1	0
Number of Genes	27,034	5,162	2,153	2,254	1,815	581	6

The number of genes present in each sub-division of inbreds. Brohammer et al. (2018) identified the presence-absence variants from re-sequencing data for 62 inbreds and used a CDS coverage of 20% to define a presence-absence variant (Brohammer *et al.*, 2018).
