# Supplementary material for: An updated gene atlas for maize reveals organ‐specific and stress‐induced genes
Source: Plant J. 2019 Jan 22;97(6):1154–67. doi: 10.1111/tpj.14184 (PMC6850026; doi:10.1111/tpj.14184)
Supplement: Supplementary file 7 — Table S2. Weighted gene co‐expression network analysis module composition [file TPJ-97-1154-s007.pdf]

Table S2: Weighted Gene Co-Expression Network Analysis Module Composition

Module	Internode	Leaf	Reproductive	Root	Seed	Abiotic	Biotic	Both Stresses	Total
1	0	0	59*	1	0	36	8	4	213
2	1	0	7	0	7	736*	65	87	2,223
3	0	1	11	625*	0	513	242*	143	2,093
4	0	0	0	0	276*	63	18	9	374
5	0	1	0	0	1	36*	0	4	43
6	40*	202	30	107	28	1207	633*	527*	4,836
7	0	0	0	0	0	9	1	1	107
8	0	0	0	0	111*	15	1	3	169
9	0	3	0	1	0	8	14*	27*	52
10	0	0	0	0	124*	71	17	13	224
11	2	1,356*	5	0	0	536	226	184	2,374
12	1	2	359*	2	3	129	35	17	882

The number of organ-specific and stress-induced differentially expressed genes in each co-expression module with the total number of genes indicated. Cells with a colored background indicate enrichment for the specific organ or stress type. *Modules enriched for organ-specific or stress-induced DE genes with an adjusted p-value < 0.001.
